# Dietary Intake of Vitamin D from Dairy Products Reduces the Risk of Osteoporosis

**DOI:** 10.3390/nu12061743

**Published:** 2020-06-10

**Authors:** Valeria Polzonetti, Stefania Pucciarelli, Silvia Vincenzetti, Paolo Polidori

**Affiliations:** 1 School of Biosciences and Veterinary Medicine, University of Camerino, 62032 Camerino (MC), Italy; valeria.polzonetti@unicam.it (V.P.); stefania.pucciarelli@unicam.it (S.P.); silvia.vincenzetti@unicam.it (S.V.); 2 School of Pharmacy, University of Camerino, 62032 Camerino (MC), Italy

**Keywords:** vitamin D, calcium, bone mass, osteoporosis, dairy foods, fortified foods

## Abstract

Background: Vitamin D and calcium are important dietary compounds that affect bone mass, even if other minerals (potassium, zinc, etc.) and vitamins (A, C and K) are also involved. Vitamin D and certain minerals, in fact, play an important role in calcium homeostasis and calcium absorption. Hip fracture incidence is higher in Europe and the United States, where calcium is frequently included in the human diet; while the occurrence of these fractures is lower in developing countries, where diets are often poor in calcium. This condition is named the “calcium paradox”, and may be partially explained by phosphate toxicity, which can negatively affect mineral metabolism. It is important to maintain correct dietary calcium-phosphate balance in order to have a healthy life, reducing the risk of osteoporotic fractures in older people. Vitamin D can also act as a hormone; vitamin D2 (ergocalciferol) is derived from the UV-B radiation of ergosterol, the natural vitamin D precursor detected in plants, fungi, and invertebrates. Vitamin D3 (cholecalciferol) is synthesized by sunlight exposure from 7-dehydrocholesterol, a precursor of cholesterol that can also act as provitamin D3. Dietary intake of vitamin D3 is essential when the skin is exposed for short periods to ultraviolet B light (UV-B), a category of invisible light rays such as UV-A and UV-C. This can be considered the usual situation in northern latitudes during the winter season, or the typical lifestyle for older people and/or for people with very white delicate skin. The actual recommended daily intake of dietary vitamin D is strictly correlated with age, ranging from 5 μg for infants, children, teenagers, and adults—including pregnant and lactating women—to 15 μg for people over 65 years.

## 1. Introduction

Vitamins are nutrients characterized by low-molecular weight; these compounds are provided by the diet and play a crucial physiological and metabolic role [1]. Vitamins are classified into two categories based on liquid solubility [2]: water-soluble vitamins (B complex and vitamin C) and fat-soluble vitamins (A, D, E, and K). Most vitamins cannot be synthesized by humans; for this reason, they must be provided by food sources or dietary supplements [3]. Vitamins are bioactive nutrients with several health-promoting properties that strongly affect human growth and health [4]. The term ‘Vitamin D’ was coined in 1922, describing a vitamin able to promote calcium deposition [5]. Vitamin D in nature is available as ergocalciferol (vitamin D2) or cholecalciferol (vitamin D3) [6]; vitamin D2 is mainly present in plants or plant products, while vitamin D3 is normally detected in animal source foods [7].

Provitamin D3 (7-dehydrocholesterol) is converted to previtamin D3 by the action of ultraviolet radiation on the skin, especially ultraviolet B light characterized by wavelength ranging between 290–315 nm. [8]; previtamin D3 is converted into 25-hydroxy vitamin D3 (25OHD3), which in turn is transformed into 1,25 dihydroxy vitamin D3 [7]. Vitamin D3 synthesis induced by sunlight is strictly correlated with the season during the year, the time of the day, the length of exposure, skin pigmentation, and latitude; in extreme latitudes, such as beyond 35 degrees North or South, vitamin D synthesis is greatly reduced or does not occur during the winter season [9]. Moreover, skin synthesis of vitamin D decreases with increase in age; consequently, the percentage of people with deficiency of vitamin D is higher in the elderly [10]. Therefore, vitamin D3 dietary intake must increase in the elderly (Table 1), but it is not easy to fulfill this target if the diet is not abundant in vitamin D-rich foods. Fatty fish, fish liver oils, and egg yolk represent the most important natural dietary sources of vitamin D [11], but these foods are not common for a large number of the consumers [12]. In meat and offals (see Table 2), vitamin D content is normally low [13]. The concentration of vitamin D in meat and liver is strictly correlated to the vitamin D content in the feed [14].

Vitamin D, both provided by food or produced by cutaneous synthesis, undergoes hydroxylation in the liver to 25-hydroxyvitamin D [25(OH)D], which is the most abundant circulating form [16]. Later, in the kidney, 25(OH)D is converted into 1,25-dihydroxy vitamin D, which is strictly correlated to calcium and phosphate absorption metabolism in the intestine [17], also influencing bone cells [18]. Parathyroid hormone has a direct effect on the production of 1,25-dihydroxy vitamin D, with specific control of the physiologic reactions necessary to link active vitamin D to calcium homeostasis [19].

The best method to determine in human body vitamin D level is represented by determination of serum concentration of 25(OH)D [20]; the optimal level for either skeletal or extra-skeletal health is not the same for everybody, but is correlated with the specific population tested.

In the human body, ingested vitamin D2 and endogenously produced vitamin D3 are converted to the biologically active form, 1,25-dihydroxyvitamin D [1,25(OH)2D], named calcitriol. In 1969, the nuclear vitamin D receptor (VDR) for 1,25(OH)2D was detected, which has to date been determined in at least 38 human tissues and organs [21]. In fact, VDR was first detected in the bone, kidney, and gastrointestinal tract; and later found in several other tissues, including those in the brain, breast, colon, and prostate [21]. Phosphoprotein VDR is involved in different biological functions of calcitriol. Because of its widespread distribution, Vitamin D is not considered just a calcaemic hormone, and lack of vitamin D is actually associated with several other diseases [22], such as psoriasis, multiple sclerosis, inflammatory bowel disease, diabetes (both type 1 and 2), hypertension, cardiovascular disease, metabolic syndromes, and different kinds of cancer [21].

Severe lack of vitamin D in adults can cause the development of osteomalacia, a disease characterized by the incomplete mineralization of osteoid [23], while in children, it is responsible for rickets [24]. Vitamin D has always been named the “antirachitic factor” [22]. Rickets is a disease characterized by decreased mineralization of bone tissue and growth plates, causing weak bones in infants and children [5]. Severe vitamin D deficiency (25 OHD < 12.5 nmol/L and levels < 25 nmol/L over a long period) in both infants and children can cause rickets, a disease where bones are deformed [25].

Chronic lack of vitamin D dietary intake is the cause of secondary hyperparathyroidism, which is a cause for increased bone turnover, with consequent progressive bone loss and finally an increased risk of bones fracture [26]. Several clinical trials have been performed on older patients to evaluate whether vitamin D supplements can decrease the incidence of fractures [27]. The results obtained in clinical trials have shown (see Table 3) a decrease in fracture incidence in patients receiving vitamin D supplementation [27].

Vitamin D supplementation, with or without calcium, can increase bone mineral density (BMD), decrease bone turnover, and decrease fracture incidence [28]. Appropriate doses of Vitamin D may differ among patients: different genetic polymorphisms, eventual presence of other diseases, and possible use of drugs can affect vitamin D metabolism [29].

## 2. Osteoporosis

Osteoporosis is a progressive disease caused by the deterioration of the bone structure because of loss in bone mineral density (BMD) [27]; its main effect is an increase in the risk of fractures [30]. In the United States, more than 40 million adults over 50 years of age are at a high risk of developing osteoporosis because of low BMD; the total number of patients affected by osteoporosis in the United States is about 12 million [19]. In Italy, 3.5 million women and 1 million men are affected by osteoporosis; each year, 250,000 fractures due to osteoporosis are reported, specifically 80,000 hip and 70,000 femur fractures [19]. Each year, patients with fracture of the proximal femur show a mortality rate of 15–30% [19]. The occurrence of osteoporosis in African-Americans is lower compared to the white population, while small white women, according to epidemiological available data, are the most affected human category [31].

“Calcium paradox” is described by the World Health Organization as the fact that countries with the highest prevalence of osteoporosis are usually those that have a high intake of calcium [32]. Bovine milk consumed in these countries contains high levels of both calcium and phosphorus; human milk phosphorus content is about 60% lower [33]. The high phosphorus content in bovine milk is necessary for proper growth of calves; however, in humans, with poor calcium bioavailability, it affects the serum calcium–phosphorus balance, triggering the parathyroid hormone to release calcium from bones. The higher protein content in bovine milk can also negatively affect the calcium balance; the final result is that the high consumption of dairy products, together with other dietary sources of phosphorus, can increase the risk of osteoporosis [32].

Low BMD characterizes osteoporosis, with consequent deterioration of the microarchitecture with trabeculae smallness, associated with reduced mineralization, and an increase in cortical porosity [34]. BMD decrease is faster during winter compared to summer [35]. Supplementing with Vitamin D (800 IU/day) combined with calcium can restrict the fall of BMD during winter [36]. A clinical trial showed a 37% lower risk of osteoporotic fracture in postmenopausal women younger than 65 when their diet was supplemented with vitamin D at a dose of 12.5 μg/day, compared to women receiving less than 3.5 μg/day vitamin D [37]. Furthermore, three other clinical trials showed that a combination of calcium and 20 μg vitamin D reduced fracture risk in adults over 65 years [35].

Vitamin D status is related to bone mineral density (BMD) not only in vitamin D deficient consumers, but also in vitamin D insufficient subjects [27]. A clinical trial on vitamin D status and BMD in 7441 postmenopausal women with osteoporosis showed a significant positive correlation between serum 25(OH)D and BMD in the trochanteric area of the hip with a threshold below 50 nmol/L [38].

Patients with osteoporosis are usually treated with bisphosphonates, calcium, and vitamin D [27]. In Italy, 1515 women with postmenopausal osteoporosis treated with bisphosphonates were classified as vitamin D deficient or vitamin D replete; the mean BMD increase per year in the lumbar spine was 0.22% in vitamin D deficient patients versus 2.11% in vitamin D replete patients; similar differences were determined in the hip [39]. These results show that the addition of both vitamin D and calcium in anti-osteoporotic treatment is necessary, unless the patient is vitamin D replete (serum 25(OH)D > 50 nmol/L) and has a dietary calcium intake of 1200 mg/d [28].

The guidelines for the management of postmenopausal osteoporosis describe the importance of satisfying vitamin D requirements in order to obtain the best response for BMD, offering recommendations for its supplementation [31], together with calcium [40]. Diet seems to moderately affect osteoporosis, but calcium and vitamin D intake is very important, at least in older patients. Diets low in dairy products have been associated with an increased risk of osteoporosis [41]. A meta-analysis of nine studies reported lower BMD of the spine and hip in vegans compared to consumers who drink milk [42].

## 3. Vitamin D and Cancer

More than 8000 studies have been performed to investigate the inverse correlation between vitamin D, its metabolites, and cancer [43]. Women with a higher solar UVB exposure in the Third National Health and Nutrition Examination Survey (NHANES III) showed 50% incidence of breast cancer compared to those with lower solar exposure [44]. In another survey, men with a higher solar UVB exposure showed only half the incidence rate of fatal prostate cancer [45]. The ultraviolet-B (UVB)–vitamin D–cancer hypothesis was based on a geographical ecological study of colon cancer mortality rates in the United States in correlation to annual sunlight exposure [46].

Meta-analyses show significant inverse correlations between serum 25(OH)D concentration and incidence of bladder, breast, colorectal, kidney, and lung cancer [43]. Two studies were reported for colon and rectal cancer. In the first one, an inverse correlation was found between 25(OH)D concentration and incidence of distal colon cancer and rectal cancer. In the second study, colon cancer cases were directly correlated with 25(OH)D concentration, while no correlation was determined for rectal cancer [47].

Another method to assess vitamin D’s role in cancer is to analyze the different cancer survival rates between black and white Americans. In the period of 2001–2004, black Americans older than 40 y had mean 25(OH)D concentrations between 35 and 43 nmol/L, while white Americans had mean concentrations around 63–65 nmo/L [48]. According to these data, black Americans would have 60% higher cancer mortality rates than white Americans. In the literature, disparities are evident for 13 cancers: bladder, breast, colon, endometrial, lung, ovarian, pancreatic, prostate, rectal, testicular, and vaginal cancer; Hodgkin’s lymphoma; and melanoma [43]. Cancer-specific mortality rates are about 25% higher for black Americans compared to white Americans.

Many other health benefits are correlated with higher 25(OH)D concentrations, including reduced risk of autoimmune diseases [49], diabetes mellitus type 2 [50], adverse pregnancy and birth outcomes [51], respiratory tract infections [52], and Celiac Disease [53]. Recently vitamin D supplementation was tested for its role in preventing coronavirus infections [54]. Thus, the increase in 25(OH)D concentrations with the aim of decreasing cancer risk will have other additional benefits [55]. The optimal 25(OH)D concentration is certainly above 75 nmol/L, probably close to 100–150 nmol/L; to obtain those concentrations, 1000–5000 IU/d of vitamin D3 is necessary, or a moderate amount of sun exposure [55]. The only way to surely determine that the desired concentration has been obtained is to evaluate serum 25(OH)D concentration [56].

The UVB–vitamin D–cancer hypothesis has considerable supporting scientific evidence from several experimental studies [55]: geographical ecological, observational, laboratory experiments, as well as many clinical trials. Lots of sun exposure and use of vitamin D3 can help prevent and treat many cancers [55]. Vitamin D supplementation can reduce the risk of cancer incidence: this thesis should be further investigated in clinical trials in order to determine the right doses of vitamin D and serum 25(OH)D concentrations, and the possible involvement of other safety issues.

## 4. Vitamin D in Dairy Products

Dairy cow breeding started around 5000 years ago during the late Neolithic and early Bronze Age in northern and central Europe [57]. “Milk” is normally associated with cow milk; however, milk from other animal species is also consumed [58].

Milk is a complete food providing several nutrients, specifically carbohydrates (mainly lactose), proteins, fat, minerals, and vitamins, contributing a mean daily intake of 134 kcal, 8 g of proteins, and 7.3 g of fat to the average human diet [59]. Water is the most represented compound in all different milks, ranging from water content lower than 50% in whale milk to water content of around 90% in donkey milk [60].

Milk is a natural source of calcium and vitamin D; these nutrients have a synergic interaction in the human body [61]. If the level of ionized calcium in the blood falls, the parathyroid hormone is secreted by the parathyroid gland, stimulating the conversion of vitamin D to its active form, calcitriol (1,25-dihydroxyvitamin D) with a consequent decrease in vitamin D status, determined by measuring the amount of the inactive form. Vitamin D, as calcitriol, influences calcium absorption in the intestine, and lack of vitamin D is associated with a reduced absorption of dietary calcium [62].

Dietary intake of vitamin D through dairy products, first of all obviously milk, has been investigated in-depth over the last 60 years [1]. In the 1960s, vitamin D content in cow milk was determined to be in the range of 0.125–1 g/L [63], while a value of 240 IU per liter was detected in cow milk for vitamin D activity, 85% of which is water soluble, attributed to vitamin D3-sulphate [64]. Vitamin D content can be described using different units: micrograms (μg) or International Units (IU); the most common unit used in Europe to describe vitamin D content is μg, while to convert μg to IU, the content in μg must be multiplied by 40 [21].

Human milk contains 24,25-dihydroxycholecalciferol and 1,25-dihydroxy vitamin D3; vitamin D content in human milk is considered very low [64]. A study on 198 children, followed up to 9 years of age, evaluated the effect of maternal vitamin D status during pregnancy on childhood skeletal growth [15]. Results obtained in 9-year-old children fed by mothers who had vitamin D insufficiency (25 OHD levels < 40 nmol/L, 31%, *n* = 49) or vitamin D deficiency (25 OHD levels < 25 nmol/L, 18%, *n* = 28) during late pregnancy showed lower whole body and lumbar spine bone mineral content (BMC). According to the results of this study, vitamin D supplementation is recommended in pregnant women, especially in the winter months, when sunlight is less. The most important result obtained is the long-lasting positive effect on peak bone mass (PBM) attainment, together with a reduced risk of osteoporotic fracture in older patients [65].

A recent study [66] determined total vitamin D content in donkey milk (23 mg/L, about 920 IU/L); it was found to be higher compared to the values obtained by analyzing milk produced by several mammalian species, including human milk [67]. Even if donkey milk represents a niche product, its use is recommended for consumers at risk of nutritional deficiencies, such as children and/or elderly; in these patients, donkey milk could help prevent lack of vitamin D [58].

Milk consumption has decreased in recent years, and dietary intake of vitamin D by way of fresh milk has declined [68], while cheese consumption has significantly increased (by almost 100%) since 1980 [68]. The big increase in human population and change in food consumption habits has created the right conditions for production of new fortified foods able to provide the recommended intake of vitamin D in the human diet. Milk does not provide the dietary requirements of vitamin D (Table 4), while cheese represents the right kind of food for the recommended dietary intake of this nutrient; in the States, the fortification level of vitamin D in cheese is strictly regulated by the U.S. Food and Drug Administration [69].

In patients with osteoporosis, treatment with drugs is the best approach to decreasing the risk of other fractures. However, even in these patients, the importance of nutrition should be taken into consideration, because inadequate intake of Ca, vitamin D, and proteins may reduce the efficacy of anti-osteoporotic drugs [69]. In one study, 37 elderly women with vitamin D deficiency received fortified soft plain cheese [69]; the dairy product provided 17–25% of the recommended dose of vitamin D (10–15 mg) and 25% for both Ca (1200 mg) and proteins (1 g/kg body weight). With daily consumption of two servings of soft plain cheese for one month, the vitamin D supplement caused a small increase in serum 25(OH)D. The results obtained in this clinical trial demonstrated that fortified soft plain cheese consumed by elderly women with vitamin D deficiency can reduce bone resorption, positively affecting Ca and protein metabolism, analyzing the decrease in PTH and increase in IGF-I, respectively [69].

## 5. Fortified Foods

Vitamin D deficiency is a public health issue that affects both men and women, causing huge human and financial costs [70]. Consumers can improve and maintain vitamin D status through increased consumption of natural or fortified food sources or vitamin D-containing dietary supplements [71]. There are only few foods naturally rich in vitamin D, and most of these show great seasonal variation in vitamin D content [72]. Frequent fish consumption can be a good strategy to maintain the required levels of 25(OH)D, as determined in elderly Japanese women [73]. Frequent fish eaters can maintain required serum 25(OH)D levels also during winter. Mushrooms represent another food that is a natural source of vitamin D; all edible mushrooms are rich of ergosterol, which, when irradiated with sunlight or UVB light, is transformed into vitamin D2 [74].

Fortification of common foods represents an easy and practical method to avoid micronutrient deficiency [75]. The first fortified food was created around 4000 years BC by the Persian physician Melampus, who enriched wine with iron filings to increase sailors’ resistance and their sexual activity [76]. Around six thousand years later, in 1833, the chemist Boussingault in France added iodine to salt to prevent goiter. In the 1920s, in Denmark, vitamin A was used to enrich margarine, while in the 1930s dairy companies in the United States began to enrich milk with vitamin D to prevent rickets in children [77].

In Canada, vitamin D fortification is compulsory for milk and margarine, while vitamin D addition to food in the U.S. is optional, with the exception of fortified milk [78]. Milk is the main fortified food in the U.S. and in Canada [72], but the amount of vitamin D added to milk (1 mg/100 g fluid milk) is not adequate to produce the desired increase or maintain circulating 25(OH)D. Vitamin D fortification at higher levels (10 mg/50 g powdered milk) showed significant effects in improving vitamin D status and bone mineralization in older women milk [79].

Milk products are systematically, either mandatorily or voluntarily, fortified with vitamin D only in Finland, Norway, Sweden, Canada, and the United States [80]. In Finland, the actual recommended fortification level of all fluid milks, with the exception of some organic products, is 1 μg/100 g, but there are also some kinds of milk marketed with a concentration of 2 μg/100 g [80]. The fortification is not compulsory, but all dairy companies are actually following these recommendations. In Norway, only one type of milk is recommended to be fortified with vitamin D at a concentration of 0.4 μg/100 g [80]. Sweden recently doubled the fortification levels of fluid milks to 1 μg/100 g and imposed a compulsory fortification for all fluid milks with less than 3% of fat [9,10].

In other countries, such as United Kingdom, Ireland, Spain, and Australia, fortification is not mandatory, but there are several vitamin D-fortified milk products available [81]. In countries where vitamin D fortification policies are strongly applied (Finland, Canada, United States), the contribution of milk to total vitamin D intake is higher compared to countries without wide fortification policies, such as Ireland, United Kingdom, Spain, and Australia [80]. The consumption of vitamin D-fortified milk was positively associated with 25(OH)D status [80], without a specific effect of local vitamin D-fortification policies. Even when the total amount of milk consumption was quite different, the correlation among milk consumption and 25(OH)D status was determined at relatively low consumption levels [82].

Vitamin D fortification of foodstuff has been shown to be a valid way to increase vitamin D dietary intake; and vitamin D-fortified fluid milk affect both vitamin D intake and 25(OH)D status [83]. However, fortification of fluid milks may not be the only strategy. Specific staple foods should be chosen in each different country as the best vitamin D carriers considering the results of simulation studies. In many countries where a fortification policy is not currently applied (see Table 5), the hypothesis of systematic vitamin D foods fortification has been considered, and simulation studies have been recently performed [84].

The optimal vitamin D status has not been determined yet; the Endocrine Society’s Clinical Practice Guidelines established a lower serum threshold for 25(OH)D level as 75 nM or 30 ng/mL [85]. The Institute of Medicine (IOM) determined these thresholds for S-25(OH)D status: <30 nmol/L is vitamin D deficient, 30–49.9 nmol/L is insufficient, and >50 nmol/L is sufficient [86].

## 6. Vitamin D Fortification Strategies

Vitamin D3, administered through cod liver oil, has been used in infant nutrition in northern Europe since the 1700s—at a daily dosage of a small teaspoon [87]. This experimental dose of cod liver oil was really effective, as discovered in studies performed two centuries later [88]; the 375 IU (9 μg) of vitamin D3 in one teaspoon was confirmed as being the most appropriate for children [89].

Vitamin D intake has been better characterized in children nutrition compared to adult requirements [90]. In the 1960s, a scientific committee determined the requirement for vitamin D in adults and recommended one-half of that was infants [87]. Strategies to improve vitamin D status in the population are based on an increased intake of naturally vitamin D containing foods, production of fortified foods, using vitamin D supplements, increasing solar UV-B exposure, and weight loss [91]. Vitamin D food fortification seems to be the best strategy to improve vitamin D intake and status in human population in order to meet dietary vitamin D recommendations [92]. Considering the lack of natural vitamin D-rich foods, some countries, particularly populations at high latitudes, developed national policies of fortifying certain foods with vitamin D to prevent deficiency of this nutrient [93]. The most common vitamin D-fortified foods are low-fat milk, fat spreads, breakfast cereals, and certain baby foods [83]. Considering the different food habits among different populations, wider vitamin D fortification of several products rather than concentrating on the limited production of a small amount of staple foods has been suggested [83]. In general, food can be enriched with vitamin D by simply adding vitamin D to it (i.e., traditional vitamin D food fortification) or practising the so called “bioaddition” [94]. Bioaddition of vitamin D, which has also been called “biofortification,” refers to different methods of increasing vitamin D content in food without direct exogenous addition of this compound [94].

Milk fortification with vitamin D started in the USA in the 1930s [70]. Milk was initially fortified using two different methods: by irradiating milk with vitamin D or feeding the cows irradiated yeast [70]. In the 1940s, a simple and valid method based on direct supplementation of vitamin D concentrate to milk was developed; it is still in use today [70].

In the United States, several RTE (ready-to-eat) breakfast cereals are fortified with vitamin D and also added to yogurt and margarines, while in Canada, it is not permitted to fortify RTE breakfast cereals. However, in fortified foods, the level of vitamin D must not exceed 20 IU/100 calories [95]. The efficacy of vitamin D food fortification in increasing vitamin D serum level has been tested [70]. Foods fortified with vitamin D normally contain 100 IU per serving; consumption of fortified milk increased vitamin D intake and was responsible for a significant increase of 25(OH)D levels [21]. An average daily intake of about 11 μg (440 IU/day) using fortified foods (range 120–1000 IU/day) achieved 25(OH)D concentrations up to 7.7 ng/mL. A daily increase of 0.5 ng/mL in 25(OH)D levels was achieved for each 40 IU (1 μg) ingested [70]. The most common food fortified with vitamin D is fresh milk, contributing 44% of the total daily vitamin D intake. Male teenagers (13 to 18 years) [82] had the highest vitamin D intake among the age/sex categories; however, on evaluating the other consumers involved in the study, dietary intake of 400 IU was not sufficient to reach serum levels of 16 ng/mL [96]. Therefore, considering that the vitamin D dietary requirement can be satisfied when in serum vitamin D level is ≥30 ng/mL, actual consumers mean intake of vitamin D can be considered low and not adequate, compared to daily nutritional requirements [97]. Higher levels of vitamin D fortification are required in order to increase the number of consumers with serum levels of 25(OH)D ≥ 20 ng/mL [98]. Vitamin D fortification strategies have also been evaluated in consumers living in developing countries, adding both vitamin D and vitamin A to fresh milk, cheese, and margarine; however, the results obtained in these trials have not been clearly discussed [99].

When considering vitamin D food fortification, it is important to evaluate whether such a public health intervention is likely to be cost-effective [100]. Usually, micronutrient fortification is considered the most cost-effective public health intervention [101]. With reference to vitamin D food fortification, there are only a few reports available on its cost-effectiveness. The available studies have reported that systematic vitamin D fortification may indeed be highly cost-effective [102]. The following distribution of costs for a typical food fortification programme was determined as follows: 80% recurrent production costs, 8% marketing and education costs, 7% food control and monitoring costs, and 5% other production costs [103].

## 7. Vitamin D Supplementation to Prevent Osteoporosis

The deposition of bone minerals begins during pregnancy, particularly in the last three months; bone mass can increase about 40 times from birth to adulthood, with a peak close to 90% occurring around the age ranging between 18 and 20 years [15]. In fact, the most critical periods for bone minerals deposition are represented by childhood and adolescence [104]. Bone is a living tissue continuously subjected to cycles of bone formation and bone resorption: poor skeletal integrity causes an increased risk of osteoporotic fractures [105].

Clinical trials have shown that mild vitamin D insufficiency can have a negative effect on bone mineral mass in adolescent females [106] and children [107]. The effect of vitamin D supplementation (200 IU/day or 400 IU/day) on bone mineral deposition has been examined in 212 adolescent girls (mean age 11.4 years) who were receiving Ca supplementation as well [108]. The results showed that bone mineral augmentation of the femur was 14.3% and 17.2% higher, respectively, in groups receiving vitamin D supplementations compared to a placebo. Furthermore, vitamin D supplementation significantly reduced bone resorption, evaluated by determining urinary deoxy-pyridinoline excretion [108]

In postmenopausal women, several studies based on vitamin D and calcium supplementation have been conducted in order to determine the best nutritional strategies [109]. A pooled analysis, describing the effect of vitamin D supplementation on fracture reduction, showed that there was a significant reduction in the incidence of hip fractures when doses higher than 792 IU/day were administered [110]. However, there was no significant decrease in hip fracture risk caused by calcium intake [111].

Circulating 25(OH)D is generally considered the most reliable marker of vitamin D status [70]. The serum content of 25(OH)D necessary to maintain adequate levels of PTH is considered to be between 30 and 100 nmol/L [112]. Because of this high variability, vitamin D insufficiency within populations can be differently evaluated depending on the threshold used. In a study performed using 8532 postmenopausal, osteoporotic European women, 79.6% were considered to found to have inadequate levels of vitamin D if the serum 25(OH)D threshold was fixed to the value of 80 nmol/L, while when the threshold was reduced to 50 nmol/L, women with severe lack of vitamin D were a smaller amount, 32.1% [113]. Based on the results obtained in several clinical trials, 80 nmol/L is considered to be an overestimated threshold, while 50 nmol/L is believed to be an acceptable threshold [109].

The dose used for vitamin D supplementation should be enough to reach the threshold values of serum 25(OH)D, otherwise the expected target will not be obtained. Clinical trials performed with the aim of determining the anti-fracture efficacy of different doses of vitamin D found that 400 IU per day was not enough to achieve a significant effect in reducing fracture rate [114]. Oral daily doses of 700–800 IU or a dose of 100,000 IU taken quarterly both showed a positive anti-fracture effect, while an annual intramuscular dose of 300,000 IU did not show valid efficacy [114]. The results obtained in these studies show that the most effective vitamin D supplementation in osteoporotic patients is obtained when administered orally either daily or quarterly; in case of a daily supplementation, the dose should be higher than 700–800 IU/day [114].

However, it is important to consider that, according to several clinical trials performed all over the world, the most effective anti-osteoporotic results were achieved with combined treatment with calcium and vitamin D supplementation [115]. In women over 65 years of age, the risk of osteoporotic fracture can be frequent, particularly if a lack of calcium is associated with vitamin D deficiency [70]. In these cases, calcium and vitamin D supplementation can be useful, administering doses of 1000–1200 mg of calcium and 800 IU of vitamin D daily, respectively [116]. The recommended strategy is to combine vitamin D and calcium into a unique supplement in order to increase a patient’s healthy status, with a consequent improvement in treatment efficacy.

## 8. Conclusions

The risk of osteoporotic fracture is increased with vitamin D deficiency [117]. In fact, biologically active vitamin D enhances calcium intestinal absorption by regulating calcium transport proteins in the small intestine, stimulating osteoclastic maturation and helping bone growth [53].

Vitamin D supplementation is required in order to reach 25(OH)D concentrations above 30 ng/mL in a large number of people. Vitamin D fortification of basic foods such as dairy and flour products can increase serum 25(OH)D concentrations, reducing the risk of osteoporosis. Prevention is absolutely necessary, considering that in 2030, 25% of the human population will be over 65 years of age.

Using appropriate feeding strategies in dairy cows, natural vitamin D content in dairy products, especially fresh milk, can be increased; further studies are necessary to optimize the total natural vitamin D content in dairy products, considering that in several countries, fortification of food is not always permitted as a common practice.

## Figures and Tables

**Table 1:** Dietary recommendations for vitamin D.

Age	Nutrient Intake (μg/Day)	Nutrient Intake (IU/Day)
0–3 months	8.5	340
4–6 months	8.5	340
7–9 months	7	280
10–12 months	7	280
1–3 years	7	280
>65 years	10	400
Pregnancy	10	400
Lactation	10	400

Source: Modified from Lanharn-New et al. [15].

**Table 2:** Vitamin D3 and Calcidiol (25(OH)-D-3) content in meat and offal.

Foodstuff	Vitamin D3 (μg/kg)	25(OH)-D-3 (μg/kg)
Beef steak	<0.5	0.8
Beef liver	<0.5	3.4
Beef kidney	1.3	3.0
Pork fillet	1.1	<0.6
Pork liver	4.0	4.4
Lamb leg steak	0.4	10.4
Chicken leg	3.0	<2.0
Chicken fillet	2.0	<2.0

Source: Modified from Lanharn-New et al. [15].

**Table 3:** Fracture risk reduction in patients receiving vitamin D supplementation.

Patients	Vitamin D Dose	Obtained 25(OH)D nmol/L	Fracture Risk Reduction
3270	800 IU/d	71	Hip: −43%
799	150,000–300,000 IU/yr	Not detected	Fractures: −24%
2686	100,000 IU/4 times per day	74	Non-vertebral fractures: −22%
9605	400 IU/d	47	Non-vertebral fractures: −16%
3195	800 IU/d	75	Fractures: −13%

Source: Modified from Lips & Van Schoof [27].

**Table 4:** Natural vitamin D content (μg/100 g) in food.

Foodstuff	Vitamin D
Whole milk	0.1
Cheese, cheddar	0.3–0.6
Yogurt	0.1
Butter	1.5
Egg yolk	4.9–5.4
Mushrooms, chanterelle	5.3–14.2
Cod liver oil	210–250
Salmon, wild	13.1–24.7
Salmon, farmed	6.0
Herring	5.7–15.4
Cod	Trace-2.6
Sole	Trace-2.8

Source: Modified from O’Mahoney et al. [21].

**Table 5:** Prevalence of Vitamin D deficiency (25-OH-D3 < 50 nmol/L) in Southeast Asia.

Country	Age (Years)	Prevalence (%)
Vietnam	Childbearing age	7
Indonesia	18–40	63
Thailand	15–98	5.7
Malaysia	7–12	72.4
Malaysia	48–53	41 males, 87 females

Source: Modified from Yang et al. [84].

## References

[1] Perales S., Alegría A., Barberá R., Farré R. (2005). Review: Determination of vitamin D in dairy products by high performance liquid chromatography. Food Sci. Technol. Int..

[2] Vincenzetti S., Astolfi G., Cellini E., Ariani A., Pucciarelli A., Cammertoni N., Polidori P. (2017). Determination of some water-soluble vitamins in donkey milk. Ital. J. Anim. Sci..

[3] Bolland M.J., Grey A., Avenell A. (2018). Effects of vitamin D supplementation on musculoskeletal health: A systematic review, meta-analysis, and trial sequential analysis. Lancet Diabetes Endocrinol..

[4] Borella E., Nesher G., Israeli E., Shoenfeld Y. (2014). Vitamin D: A new anti-infective agent?. Ann. N.Y. Acad. Sci..

[5] O’Riordan J.L., Bijvoet O.L. (2014). Rickets before the discovery of vitamin D. Bonekey Rep..

[6] Wacker M., Holick M.F. (2013). Vitamin D—Effects on skeletal and extraskeletal health and the need for supplementation. Nutrients.

[7] Barvencik F., Amling M. (2015). Vitamin D metabolism of the bone. Orthopade.

[8] Við Streym S., Højskov C.S., Møller U.K., Heickendorff L., Vestergaard P., Mosekilde L., Rejnmark L. (2016). Vitamin D content in human breast milk: A 9-mo follow-up study. Am. J. Clin. Nutr..

[9] Zhang R.H., He D.H., Zhou B., Zhu Y.B., Zhao D., Huang L.C., Ding G.Q. (2015). Analysis of vitamin D status in men highly exposed to sunlight. Biomed. Environ. Sci..

[10] Lips P., Hosking D., Lippuner K., Norquist J.M., Wehren L., Maalouf G., Ragi-Eis S., Chandler J. (2006). The prevalence of vitamin D inadequacy amongst women with osteoporosis: An international epidemiological investigation. J. Intern. Med..

[11] Henderson L., Gregory J., Swan G. (2002). National Diet and Nutrition Survey (NDNS): Adults Aged 19–64 Years.

[12] Lips P. (2001). Vitamin D deficiency and secondary hyperparathyroidism in the elderly: Consequences for bone loss and fractures and therapeutic implications. Endocr. Rev..

[13] Williams P. (2007). Nutritional composition of red meat. Nutr. Diet..

[14] Jakobsen J., Maribo H., Bysted A., Sommer H.M., Hels O. (2007). 25-Hydroxyvitamin D3 affects vitamin D status similar to vitamin D3 in pigs – but the meat produced has a lower content of vitamin D. Br. J. Nutr..

[15] Lanham-New S.A., Thompson R.L., More J., Brooke-Wavell K., Hunking P., Medici E. (2007). Importance of vitamin D, calcium and exercise to bone health with specific reference to children and adolescents. Nutr. Bull..

[16] Shinchuk L., Holick M.F. (2007). Vitamin D and rehabilitation: Improving functional outcomes. Nutr. Clin. Pract..

[17] Marques Garcia A.F.Q., Murakami A.E., do Amaral Duarte C.R., Ospina Rojas I.C., Picoli K.P., Mangili Puzotti M. (2013). Use of Vitamin D3 and Its Metabolites in Broiler Chicken Feed on Performance, Bone Parameters and Meat quality. Asian-Austral. J. Anim. Sci..

[18] Schmid A., Walther B. (2013). Natural vitamin D content in animal products. Adv. Nutr..

[19] Moioli C., Tagliabue L., Cioni F. (2018). Osteoporosis and mineral nutrition. A literature review. Prog. Nutr..

[20] Gill B.D., Abernethy G.A., Green R.J., Indyk H.E. (2016). Analysis of Vitamin D2 and Vitamin D3 in Fortified Milk Powders and Infant and Nutritional Formulas by Liquid Chromatography–Tandem Mass Spectrometry: Single-Laboratory Validation, First Action 2016.05. J. AOAC Int..

[21] O’Mahony L., Stepien M., Gibney M.J., Nugent A.P., Brennan L. (2011). The Potential Role of Vitamin D Enhanced Foods in Improving Vitamin D Status. Nutrients.

[22] Kiraly S.J., Kiraly M.A., Hawe R.D., Makhani N. (2006). Vitamin D as a neuroactive substance. Sci. World J..

[23] Rader C.P., Corsten N., Rolf O. (2015). Osteomalacia and vitamin D deficiency. Orthopade.

[24] Sanders K., Stuart A.L., Williamson E.J., Simpson J.A., Kotowicz M.A., Young D., Geoffrey C., Nicholson G.C. (2010). Annual high-dose oral vitamin D and falls and fractures in older women: A randomized controlled trial. J. Am. Med. Assoc..

[25] Wintermeyer E., Ihle C., Ehnert S., Stöckle U., Ochs G., de Zwart P., Flesch I., Bahrs C., Nussler A.K. (2016). Crucial Role of Vitamin D in the Musculoskeletal System. Nutrients.

[26] Ishijima M., Sakamoto Y., Yamanaka M., Tokita A., Kitahara K., Kaneko A., Kurosawa H. (2010). Minimum required vitamin D level for optimal increase in bone mineral density with alendronate treatment in osteoporotic women. Calc. Tissue Int..

[27] Lips P., van Schoor N.M. (2011). The effect of vitamin D on bone and osteoporosis. Best Pract. Res. Clin. Endocrin. Metabol..

[28] Bolland M.J., Avenell A., Baron J.A., Grey A., MacLennan G.S., Gamble G.D., Reid I.R. (2010). Effect of calcium supplements on risk of myocardial infarction and cardiovascular events: Meta-analysis. Br. Med. J..

[29] Boonen S., Lips P., Bouillon R., Bischoff-Ferrari H.A., Vanderschueren D., Haentjens P. (2007). Need for additional calcium to reduce the risk of hip fracture with vitamin d supplementation: Evidence from a comparative metaanalysis of randomized controlled trials. J. Clin. Endocrin. Metab..

[30] Rizzoli R., Boonen S., Brandi M.L., Burlet N., Delmas P., Reginster J.Y. (2008). The role of calcium and vitamin D in the management of osteoporosis. Bone.

[31] Hegsted D.M. (1986). Calcium and osteoporosis. J. Nutr..

[32] Mahdi A.A., Brown R.B., Razzaque M.S. (2015). Osteoporosis in Populations with High Calcium Intake: Does Phosphate Toxicity Explain the Paradox?. Ind. J. Clin. Biochem..

[33] Salimei E., Fantuz F., Coppola R., Chiofalo B., Polidori P., Varisco G. (2004). Composition and characteristics of ass’s milk. Anim. Res..

[34] Bone H.G., Hosking D., Devogelaer J.P., Tucci J.R., Emkey R.D., Tonino R.P., Rodriguez-Portales J.A., Downs R.W., Gupta J., Santora A.C. (2004). Ten years experience with alendronate for osteoporosis in postmenopausal women. N. Engl. J. Med..

[35] Vieth R., Feldman D., Wesley Pike J., Adams J.S. (2011). The Pharmacology of Vitamin D, Including Fortification Strategies. “Vitamin D”.

[36] Dawson-Hughes B., Dallal G.E., Krall E.A., Sokoll L.J., Falconer G. (1991). Effect of vitamin D supplementation on wintertime and overall bone loss in healthy postmenopausal women. Ann. Int. Med..

[37] Feskanich D., Willett W.C., Colditz G.A. (2003). Calcium, vitamin D, milk consumption, and hip fractures: A prospective study among postmenopausal women. Am. J. Clin. Nutr..

[38] Wenclewska S., Szymczak-Pajor I., Drzewoski J., Bunk M., Sliwìnska A. (2019). Vitamin D Supplementation Reduces Both Oxidative DNA Damage and Insulin Resistance in the Elderly with Metabolic Disorders. Int. J. Mol. Sci..

[39] Adami S., Giannini S., Bianchi G., Sinigaglia L., Di Munno O., Fiore C.E., Minisola S., Rossini M. (2009). Vitamin D status and response to treatment in post-menopausal osteoporosis. Osteoporos. Int..

[40] Grant W.B. (2012). Ecological Studies of the UVB–Vitamin D–Cancer Hypothesis. Anticancer Res..

[41] Merrill R.M., Aldana S.G. (2009). Consequences of a plant-based diet with low dairy consumption on intake of bone-relevant nutrients. J. Women’s Health.

[42] Ho-Pham L.T., Nguyen N.D., Nguyen T.V. (2009). Effect of vegetarian diets on bone mineral density: A Bayesian meta-analysis. Am. J. Clin. Nutr..

[43] Grant W.B. (2018). A Review of the Evidence Supporting the Vitamin D-Cancer Prevention Hypothesis in 2017. Anticancer Res..

[44] John E.M., Schwartz G.G., Dreon D.M., Koo J. (1999). Vitamin D and breast cancer risk: The NHANES I epidemiologic follow-up study, 1971–1975 to 1992. Cancer Epidemiol. Biomark. Prev..

[45] John E.M., Schwartz G.G., Koo J., Van Den Berg D., Ingles S.A. (2005). Sun exposure, vitamin D receptor gene polymorphisms, and risk of advanced prostate cancer. Cancer Res..

[46] Garland C.F., Gorham E.D., Sharif B., Mohr S.B., Garland F.C. (2009). Vitamin D for Cancer Prevention: Global Perspective. Ann. Epidemiol..

[47] Weinstein S.J., Yu K., Horst R.L., Ashby J., Virtamo J., Albanes D. (2011). Serum 25-hydroxyvitamin d and risks of colon and rectal cancer in finnish men. Am. J. Epidemiol..

[48] Grant W.B., Peiris A.N. (2012). Differences in vitamin d status may account for unexplained disparities in cancer survival rates between african and white americans. Dermatoendocrinology.

[49] Dankers W., Colin E.M., van Hamburg J.P., Lubberts E. (2016). Vitamin d in autoimmunity: Molecular mechanisms and therapeutic potential. Front. Immunol..

[50] Berridge M.J. (2017). Vitamin d deficiency and diabetes. Biochem. J..

[51] Wagner C.L., Hollis B.W., Kotsa K., Fakhoury H., Karras S.N. (2017). Vitamin d administration during pregnancy as prevention for pregnancy, neonatal and postnatal complications. Rev. Endocr. Metab. Disord..

[52] Martineau A.R., Jolliffe D.A., Hooper R.L., Greenberg L., Aloia J.F., Bergman P., Dubnov-Raz G., Esposito S., Ganmaa D., Ginde A.A. (2017). Vitamin d supplementation to prevent acute respiratory tract infections: Systematic review and metaanalysis of individual participant data. BMJ.

[53] Vici G., Camilletti D., Polzonetti V. (2020). Possible Role of Vitamin D in Celiac Disease Onset. Nutrients.

[54] Grant W.B., Lahore H., McDonnell S.L., Baggerly C.A., French C.B., Aliano J.L., Bhattoa H.P. (2020). Evidence that Vitamin D Supplementation Could Reduce Risk of Influenza and COVID-19 Infections and Deaths. Nutrients.

[55] Jiguang M., Zhenhua M., Wei L., Qingyong M., Jian G., Ang H., Rong L., Fengfei W., Suxia H. (2013). The Mechanism of Calcitriol in Cancer Prevention and Treatment. Curr. Med. Chem..

[56] Garland C.F., French C.B., Baggerly L.L., Heaney R.P. (2011). Vitamin d supplement doses and serum 25-hydroxyvitamin d in the range associated with cancer prevention. Anticancer Res..

[57] Curry A. (2013). The milk revolution. Nature.

[58] Polidori P., Vincenzetti S. (2019). The Therapeutic, Nutritional and Cosmetic Properties of Donkey Milk.

[59] Polidori P., Ariani A., Vincenzetti S. (2015). Use of Donkey Milk in Cases of Cow’s Milk Protein Allergies. Int. J. Child Health Nutr..

[60] Vincenzetti S., Pucciarelli S., Polzonetti V., Polidori P. (2017). Role of proteins and of some bioactive peptides on the nutritional quality of donkey milk and their impact on human health. Beverages.

[61] Spence L.A., Cifelli C.J., Miller G.D. (2011). The role of dairy products in healthy weight and body composition in children and adolescents. Curr. Nutr. Food Sci..

[62] Tang B.M.P., Eslick G.D., Nowson C., Smith C., Bensoussan A. (2007). Use of calcium or calcium in combination with vitamin D supplementation to prevent fractures and bone loss in people aged 50 years and older: A meta-analysis. Lancet.

[63] Leerbeck E., Søndergaard H. (1980). The total content of vitamin D in human milk and cow’s milk. Br. J. Nutr..

[64] Reeve L.E., Jorgensen N.A., DeLuca H.F. (1982). Vitamin D compounds in cow’s milk. J. Nutr..

[65] Cooper C., Javaid K., Westlake S., Harvey N., Dennison E. (2005). Developmental origins of osteoporotic fracture: The role of maternal vitamin D insufficiency. J. Nutr..

[66] Altomonte I., Salari F., Licitra R., Martini M. (2019). Donkey and human milk: Insights into their compositional similarities. Int. Dairy J..

[67] Martini M., Altomonte I., Licitra R., Salari F. (2018). Short communication: Technological and seasonal variations of vitamin D and other nutritional components in donkey milk. J. Dairy Sci..

[68] Dimartino G. (2007). Convenient Analysis of Vitamin D in Cheese and Other Food Matrixes by Liquid Chromatography/Mass Spectrometry. J. AOAC Int..

[69] Bonjour J.P., Benoit V., Pourchaire O., Ferry M., Rousseau B., Souberbielle J.C. (2009). Inhibition of markers of bone resorption by consumption of vitamin D and calcium-fortified soft plain cheese by institutionalised elderly women. Br. J. Nutr..

[70] Black L.J., Seamans K.M., Cashman K.D., Kiely M. (2012). An updated systematic review and meta-analysis of the efficacy of vitamin D food fortification. J. Nutr..

[71] Calvo M.S., Whiting S.J. (2006). Public health strategies to overcome barriers to optimal vitamin D status in populations with special needs. J. Nutr..

[72] Calvo M.S., Whiting S.J., Barton C.N. (2005). Vitamin D intake: A global perspective of current status. J. Nutr..

[73] Nakamura K., Nashimoto M., Hori Y., Yamamoto M. (2000). Serum 25-hydroxyvitamin D concentrations and related dietary factors in peri- and postmenopausal Japanese women. Am. J. Clin. Nutr..

[74] Jasinghe V.J., Perera C.O. (2005). Distribution of ergosterol in different tissues of mushrooms and its effect on the conversion of ergosterol to vitamin D2 by UV irradiation. Food Chem..

[75] Gupta A. (2014). Vitamin D deficiency in India: Prevalence, causalities and interventions. Nutrients.

[76] Panda A.K., Mishra S., Mohapatra S.K. (2011). Iron in ayurvedic medicine. J. Adv. Dev. Res..

[77] Rajakumar K., Greenspan S.L., Thomas S.B., Holick M.F. (2007). Solar ultraviolet radiation and vitamin D: A historical perspective. Am. J. Public Health.

[78] Calvo M.S., Whiting S.J., Barton C.N. (2004). Vitamin D fortification in the US and Canada: Current status and data needs. Am. J. Clin. Nutr..

[79] Chee W.S., Suriah A.R., Chan S.P., Zaitan Y., Chan Y.M. (2003). The effect of milk supplementation on bone mineral density in postmenopausal Chinese women in Malaysia. Osteoporos. Int..

[80] Itkonen S.T., Erkkol M., Lamberg-Allardt C.J.E. (2018). Vitamin D Fortification of Fluid Milk Products and Their Contribution to Vitamin D Intake and Vitamin D Status in Observational Studies—A Review. Nutrients.

[81] Hennessy Á., Browne F., Kiely M., Walton J., Flynn A. (2017). The role of fortified foods and nutritional supplements in increasing vitamin D intake in Irish preschool children. Eur. J. Nutr..

[82] Vatanparast H., Calvo M.S., Green T.J., Whiting S.J. (2010). Despite mandatory fortification of staple foods, vitamin D intakes of canadian children and adults are inadequate. J. Steroid Biochem. Mol. Biol..

[83] Cashman K.D., Kiely M. (2016). Tackling inadequate vitamin D intakes within the population: Fortification of dairy products with vitamin D may not be enough. Endocrine.

[84] Yang Z., Laillou A., Smith G., Schofield D., Moench-Pfanner R. (2013). A review of vitamin D fortification: Implications for nutrition programming in Southeast Asia. Food Nutr. Bull..

[85] Holick M.F., Binkley N.C., Bischoff-Ferrari H.A., Gordon C.M., Hanley D.A., Heaney R.P., Murad M.H., Weaver C.M. (2012). Guidelines for preventing and treating vitamin D deficiency and insufficiency revisited. J. Clin. Endocrinol. Metab..

[86] Institute of Medicine Food and Nutrition Board (2011). Dietary Reference Intakes for Adequacy: Calcium and Vitamin D.

[87] Vieth E. (1999). Vitamin D supplementation, 25-hydroxyvitamin D concentrations, and safety. Am. J. Clin. Nutr..

[88] Zeghoud F., Vervel C., Guillozo H., Walrant-Debray O., Boutignon H., Garabedian M. (1997). Subclinical vitamin D deficiency in neonates: Definition and response to vitamin D supplements. Am. J. Clin. Nutr..

[89] Pittard W.B., Geddes K.M., Hulsey T.C., Hollis B.W. (1991). How much vitamin D for neonates?. Am. J. Dis. Child..

[90] Chesney R.W. (2001). Vitamin D deficiency and rickets. Rev. Endocr. Metab. Disord..

[91] Abrahamsen B. (2017). Bespoke or one size fits all-Vitamin D fortification, targeted supplementation in risk groups or individual measurement?. Maturitas.

[92] Hayes A., Cashman K.D. (2017). Food-based solutions for vitamin D deficiency: Putting policy into practice and the key role for research. Proc. Nutr. Soc..

[93] Lamberg-Allardt C., Brustad M., Meyer H.E., Steingrimsdottir L. (2013). Vitamin D—A systematic literature review for the 5th edition of the Nordic Nutrition Recommendations. Food Nutr. Res..

[94] Cashman K.D. (2015). Vitamin D: Dietary requirements and food fortification as a means of helping achieve adequate vitamin D status. J. Steroid Biochem. Mol. Biol..

[95] Calvo M.S., Whiting S.J. (2013). Survey of current vitamin D food fortification practices in the United States and Canada. J. Steroid Biochem. Mol. Biol..

[96] Ross A.C., Manson J.E., Abrams S.A., Aloia J.F., Brannon P.M., Clinton S.K., Durazo-Arvizu R.A., Gallagher J.C., Gallo R.L., Jones G. (2011). The 2011 dietary reference intakes for calcium and vitamin D: What dietetics practitioners need to know. J. Am. Diet Assoc..

[97] Cashman K.D. (2018). Vitamin D requirements for the future-lessons learned and charting a path forward. Nutrients.

[98] Brown J., Sandmann A., Ignatius A., Amling M., Barvencik F. (2013). New perspectives on vitamin D food fortification based on a modeling of 25(OH)D concentrations. Nutr. J..

[99] Darnton-Hill I., Darnton-Hill I., Nalubola R. (2002). Fortification strategies to meet micronutrient needs: Successes and failures. Proc. Nutr. Soc..

[100] Ethgen O., Hiligsmann M., Burlet N., Reginster J.Y. (2015). Public health impact and cost-effectiveness of dairy products supplemented with vitamin D in prevention of osteoporotic fractures. Arch. Public Health.

[101] Fiedler J.L., Sanghvi T.G., Saunders M.K. (2008). A review of the micronutrient intervention cost literature: Program design and policy lessons. Int. J. Health Plan. Manag..

[102] Hiligsmann M., Reginster J.Y. (2018). The projected public health and economic impact of vitamin D fortified dairy products for fracture prevention in France. Expert Rev. Pharm. Outcomes Res..

[103] Sandmann A., Amling M., Barvencik F., König H.H., Bleibler F. (2017). Economic evaluation of vitamin D and calcium food fortification for fracture prevention in Germany. Public Health Nutr..

[104] Di Somma C., Scarano E., Barrea L., Zhukouskaya V.V., Savastano S., Mele C., Scacchi M., Aimaretti G., Colao A., Marzullo P. (2017). Vitamin D and Neurological Diseases: An Endocrine View. Int. J. Mol. Sci..

[105] Russell A.S., Dennison E., Cooper C., New S.A., Bonjour P. (2003). Epidemiology and public health impact of osteoporosis. Nutritional Aspects of Bone Health.

[106] Outila T.A., Kakkainen M.U., Lamberg-Allardt C.J. (2001). Vitamin D status affects serum parathyroid hormone concentrations during winter in female adolescents: Associations with forearm bone mineral density. Am. J. Clin. Nutr..

[107] Cheng S., Tylavsky F., Kroger H., Kärkkäinen M., Lyytikäinen A., Koistinen A., Mahonen A., Alen M., Halleen J., Väänänen K. (2003). Association of low 25-hydroxvitamin D concentrations with elevated parathyroid hormone concentrations and low cortical bone density in early pubertal and prepubertal Finnish girls. Am. J. Clin. Nutr..

[108] Viljakainen H.T., Natri A.M., Karkkainen M., Huttunen M.M., Palssa A., Jakobsen J., Cashman K.D., Mølgaard C., Lamberg-Allard C. (2006). A positive dose–response effect of vitamin D supplementation on site-specific bone mineral augmentation in adolescent girls: A double-blinded randomized placebo-controlled 1-year intervention. J. Bone Miner. Res..

[109] Chapuy M.C., Pamphile R., Paris E., Chapuy M.C., Pamphile R., Kempf C., Schlichting M., Arnaud S., Garnero P., Meunier P.J. (2010). Combined calcium and vitamin D3 supplementation in elderly women: Confirmation of reversal of secondary hyperparathyroidism and hip fracture risk: The Decalyos II study. Osteoporos. Int..

[110] Bischoff-Ferrari H.A., Willett W.C., Orav E.J., Lips P., Meunier P.J., Lyons R.A., Flicker L., Wark J., Jackson R.D., Cauley J.A. (2012). A pooled analysis of vitamin D dose requirements for fracture prevention. N. Engl. J. Med..

[111] Specker B., Binkley T. (2003). (2003) Randomized trial of physical activity and calcium supplementation on bone mineral content in 3-to 5-year-old children. J. Bone Miner. Res..

[112] Dawson-Hughes B., Heaney R.P., Holick M.F., Lips P., Meunier P.J., Vieth R. (2005). Estimates of optimal vitamin D status. Osteoporos. Int..

[113] Bischoff-Ferrari H.A., Willett W.C., Wong J.B., Giovannucci E., Dietrich T., Dawson-Hughes B. (2005). Fracture prevention with vitamin D supplementation. JAMA.

[114] Bischoff-Ferrari H.A. (2007). How to select the doses of vitamin D supplementation in the management of osteoporosis. Osteoporos. Int..

[115] Sahota O., Mundey M.K., San P., Godber I.M., Lawson N., Hosking D.J. (2004). The relationship between vitamin D and parathyroid hormone: Calcium homeostasis, bone turnover, and bone mineral density in postmenopausal women with established osteoporosis. Bone.

[116] Bailey R.L., Dodd K.W., Goldman J.A., Cahche J.J., Dwyer J.T., Moshfegh A.J., Sempos C.T., Picciano M.F. (2010). Estimation of total usual calcium and vitamin D intakes in the United States. J. Nutr..

[117] Lips P., Bouillon R., van Schoor N.M., Vanderschueren D., Verschueren S., Kuchuk N., Milisen K., Boonen S. (2010). Reducing fracture risk with calcium and vitamin D. Clin. Endocrinol..

